# Supplementary material for: Efficacy of Fecal Sampling as a Gut Proxy in the Study of Chicken Gut Microbiota
Source: Front Microbiol. 2019 Sep 13;10:2126. doi: 10.3389/fmicb.2019.02126 (PMC6753641; doi:10.3389/fmicb.2019.02126)
Supplement: Supplementary file 1 [file Table_1.docx]

**Supplementary Table S1** Composition and ingredients of the diets for chickens during experiment

| Ingredient (%) | 1-21d | 22-42d | 43-77d |
| --- | --- | --- | --- |
| Corn | 61.99 | 66.05 | 69.29 |
| Soybean meal | 24.80 | 16.14 | 9.17 |
| Cottonseed meal（46%） | 4.00 | 6.00 | 7.90 |
| Corn protein（46%） | 3.00 | 4.00 | 5.00 |
| Soybean oil | 1.52 | 3.17 | 4.10 |
| Dicalcium phosphate | 1.86 | 1.64 | 1.47 |
| Limestone | 1.00 | 1.05 | 1.06 |
| NaCl | 0.26 | 0.21 | 0.22 |
| Choline chloride (50%) | 0.10 | 0.10 | 0.10 |
| Lysine•HCl(78%) | 0.47 | 0.64 | 0.69 |
| Premix^*^ | 1.00 | 1.00 | 1.00 |
| Total | 100.00 | 100.00 | 100.00 |
|  |  |  |  |
| Calculation of nutrients |  |  |  |
| Metabolizable energy, MJ/kg | 2900 | 3050 | 3150 |
| Crude protein, % | 20.50 | 18.50 | 17.00 |
| Calcium, % | 0.90 | 0.85 | 0.80 |
| Total phosphorus, % | 0.69 | 0.64 | 0.60 |
| Available phosphorus, % | 0.45 | 0.41 | 0.38 |
| Lysine, % | 1.10 | 1.00 | 0.88 |
| Methionine + Cystine, % | 0.84 | 0.78 | 0.65 |
| Threonine, % | 0.70 | 0.65 | 0.58 |

^*^ Provided the following % per kilogram in completed diet: vitamin A, 10,000 IU; vitamin D3, 2,500 IU; vitamin E, 46 IU; vitamin K3, 2.50 mg; vitamin B1, 5.60 mg; vitamin B2, 11 mg; vitamin B6, 8 mg; vitamin B12, 10.5 μg; nicotinic acid, 60 mg; pantothenic acid, 32 mg; folic acid, 2 mg; biotin, 22.4 μg; Fe, 80 mg; Zn, 35 mg; Mn, 60 mg; Cu, 10 mg; I, 0.42 mg; Co, 0.24 mg; and Se, 0.35 mg.
