# Supplementary material for: Efficacy of Fecal Sampling as a Gut Proxy in the Study of Chicken Gut Microbiota
Source: Front Microbiol. 2019 Sep 13;10:2126. doi: 10.3389/fmicb.2019.02126 (PMC6753641; doi:10.3389/fmicb.2019.02126)
Supplement: Supplementary file 7 [file Image_1.pdf]

SUPPLEMENTARY FIGURES

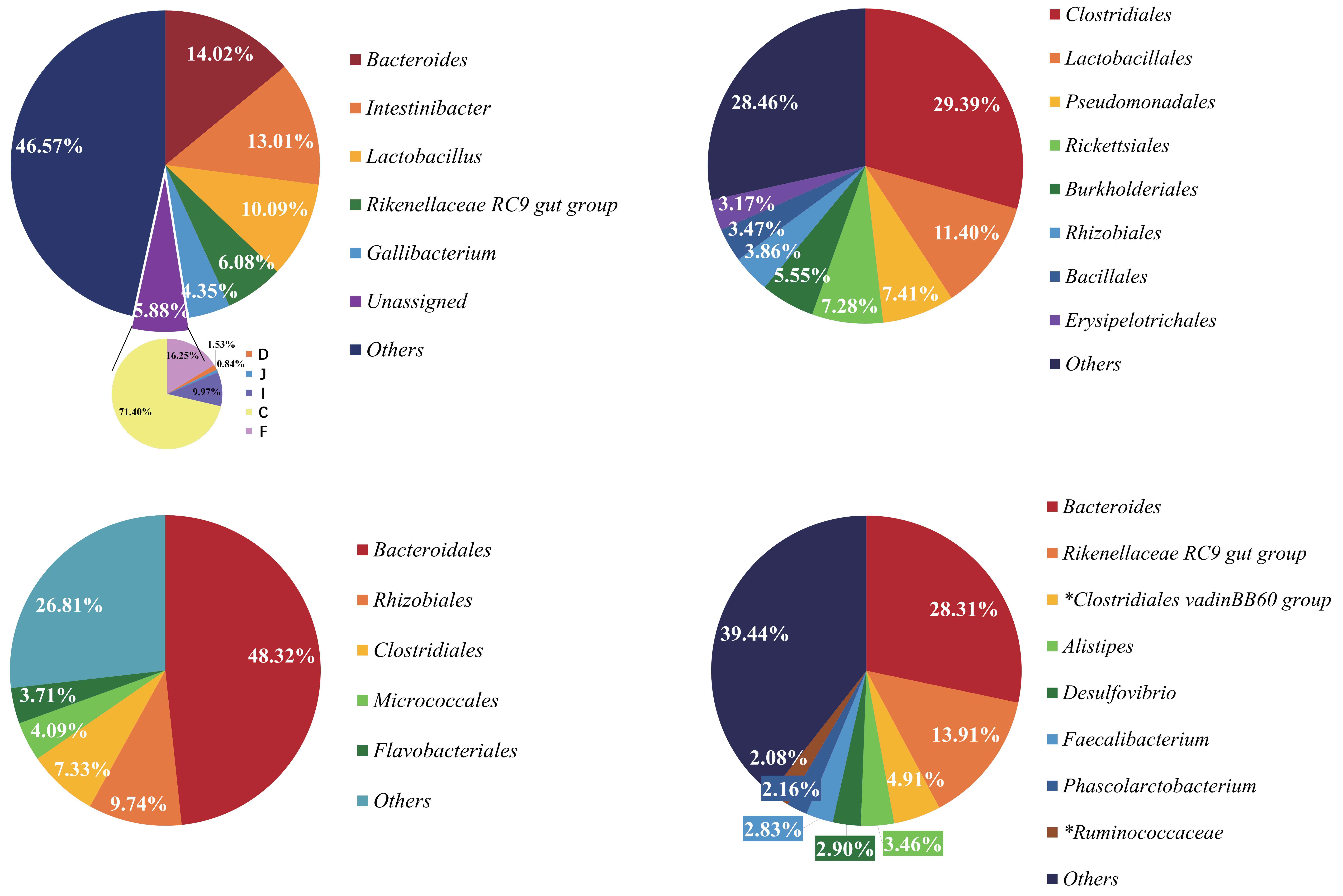

Supplementary Figure S1 The predominant taxa. (A) The dominant taxa of the core microbiota. The sequences of unassigned taxa are partitioned to different sampling sites (duodenum, jejunum, ileum, ceca and feces) shown in the small pie chart. (B) The predominant taxa exclusively shared between feces and small intestine. (C) The predominant taxa exclusively shared between feces and cecum. (D) The dominated taxa in cecum samples.
