# Supplementary material for: Efficacy of Fecal Sampling as a Gut Proxy in the Study of Chicken Gut Microbiota
Source: Front Microbiol. 2019 Sep 13;10:2126. doi: 10.3389/fmicb.2019.02126 (PMC6753641; doi:10.3389/fmicb.2019.02126)
Supplement: Supplementary file 8 [file Image_2.pdf]

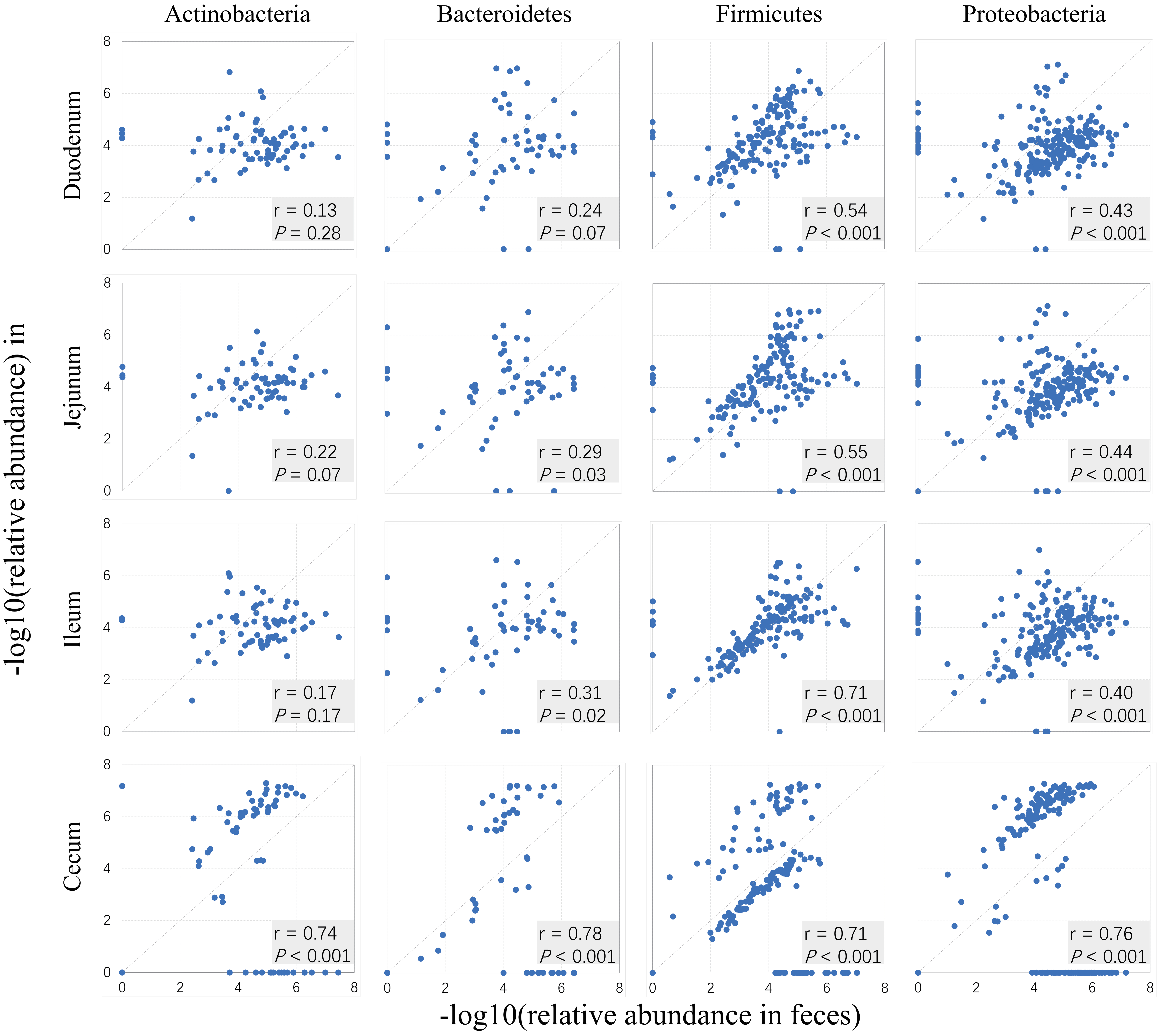

Supplementary Figure S2 Related to Figure 3: A phylum-level view of gastrointestinal (GI) taxa mirrored in feces. The four dominating phyla (Actinobacteria, Bacteroidetes, Firmicutes and Proteobacteria) are shown. Each dot represents the average relative abundance of a genus within one of the above four phyla across all individuals for each site (feces at the x-axis; duodenum, jejunum, ileum and ceca at the y-axis). Spearman's rho is calculated between fecal and GI negative logarithm-transferred relative abundances. *P*-values indicate the significance of the correlations.
