# Supplementary material for: Efficacy of Fecal Sampling as a Gut Proxy in the Study of Chicken Gut Microbiota
Source: Front Microbiol. 2019 Sep 13;10:2126. doi: 10.3389/fmicb.2019.02126 (PMC6753641; doi:10.3389/fmicb.2019.02126)
Supplement: Supplementary file 9 [file Image_3.pdf]

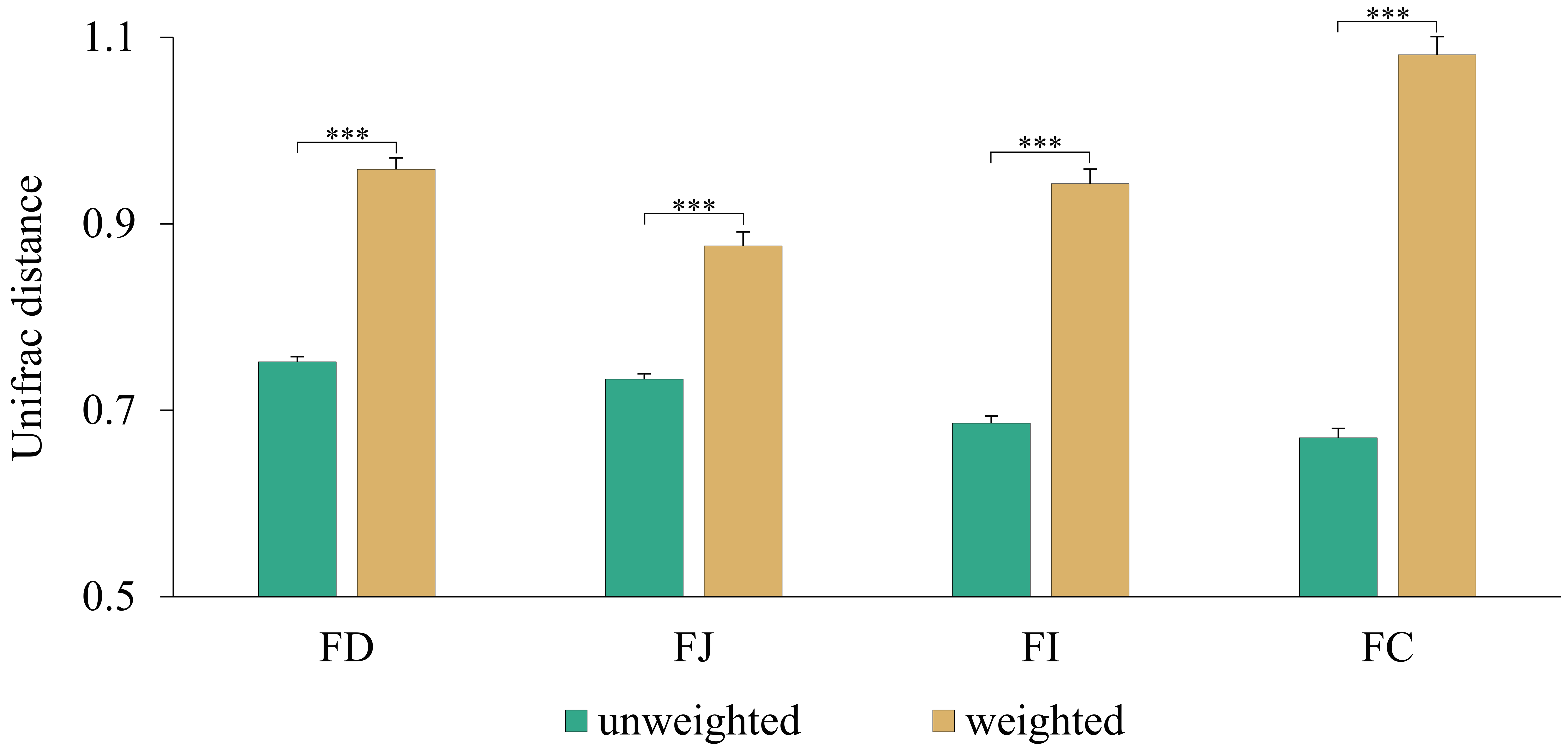

Supplementary Figure S3 Comparisons of microbial community membership and structure. D, J, I, C and F denote the microbial community of the duodenum, jejunum, ileum, cecum and feces, respectively. FD represents the UniFrac distance (mean ± SEM) between fecal and duodenal microbial communities, equivalent to FJ, FI and FC. Asterisks indicate the significance of the paired *t*-test: ***$P < 0.001$.
